# Latissimus dorsi musculocutaneous flap grafting to the infected recipient site in a patient with irradiated locally advanced breast cancer and multiple lung metastases

**DOI:** 10.1186/s40792-023-01711-x

**Published:** 2023-07-10

**Authors:** Yuki Ishii, Hitomi Matsuki, Nozomi Uozumi, Shoji Oura

**Affiliations:** Department of Surgery, Kishiwada Tokushukai Hospital, 4-27-1, Kamori-Cho, Kishiwada city, Osaka 596-8522 Japan

**Keywords:** Anti-coagulant therapy, Breast cancer, Musculocutaneous flap, Infected recipient site

## Abstract

**Background:**

Musculocutaneous (MC) flaps are more resistant to infection than implants, but no clinical results have been reported so far about the grafting of MC flap to the overtly infected sites.

**Case presentation:**

A 66-year-old woman had received radiotherapy, a total dose of 50 Gy, to her large mucinous breast cancer to control bleeding from the tumor and was referred to our hospital for further treatment. On her first visit to our hospital, her left breast showed radiation-induced total necrosis with *Pseudomonas* *aeruginosa* infection. Removal of the necrotic breast tissue resulted in direct exposure of the left ribs and intercostal muscles with intractable chest pain requiring analgesics. The presence of concomitant life-threatening multiple lung metastases made us change the treatment from letrozole and palbociclib to bevacizumab and paclitaxel, leading to marked regression of the lung metastases. To alleviate her chest pain and get local wound healing, we treated the patient with latissimus dorsi (LD)-MC flap grafting to the exposed chest wall after four months of taxane-containing chemotherapy. The patient has got marked pain relief immediately after the operation. Skin island of the grafted LD-MC flap showed no problems for 4 days just after the operation but gradually turned out to be edematous to ill-colored in the distal part of the skin island. Post-operative clinical outcome suggested that *Pseudomonas* *aeruginosa* infection might have had some adverse effect, e.g., microemboli, on MC flap blood flow. Partial necrosis of the LD-MC flap made the patient receive conservative wound management for a very long period of 11 months, finally leading to complete wound healing. After palliative surgery, the patient has been receiving fulvestrant and palbociclib for 14 months and doing well with good control of multiple lung metastases.

**Conclusions:**

Breast surgical oncologists should note that partial flap necrosis can occur when a LD-MC flap is grafted to the infected recipient site and consider the anti-coagulant therapy just after the operation to avoid the adverse effects of the infection.

## Background

In the heyday of Halsted surgery [1], routine wide skin resection just above breast cancer often forced breast surgeons to fill the large skin defect with skin grafting. However, with the earlier detection of breast cancer and the widespread use of radiotherapy to the conserved breast or chest wall [2], the extent of skin resection has gradually become smaller, and the chances to fill the skin defects with skin grafting have decreased dramatically in breast cancer surgery.

Patients with large invasive breast cancers have often come to be treated not only with mastectomy, but also with breast-conserving surgery when observed with marked concentric shrinkage of the large breast cancer by the neoadjuvant chemotherapy [3]. In contrast, patients with wide spread non-invasive, i.e., stage 0, breast cancers have long been treated not with breast conservation but either with mastectomy or total glandectomy followed by some kind of immediate breast reconstruction.

In the treatment of breast cancer, breast reconstruction mainly consists of implant-based and flap-based surgeries. The former has the advantages that it can be used for bilateral breast reconstruction and needs no flap harvesting. The latter is more resistant to infection than the former. Resistance to infection, however, is for the newly developing infection and naturally does not imply the ability to control the pre-existing infection. On the contrary, no reports have been thus far made about the clinical consequences of musculocutaneous (MC) flap grafting to the overtly infected recipient site.

We herein report an extremely rare case of metastatic breast cancer with a large infectious skin defect treated with multidisciplinary treatment including latissimus dorsi (LD)-MC flap grafting [4].

## Case presentation

**Fig. 1:**
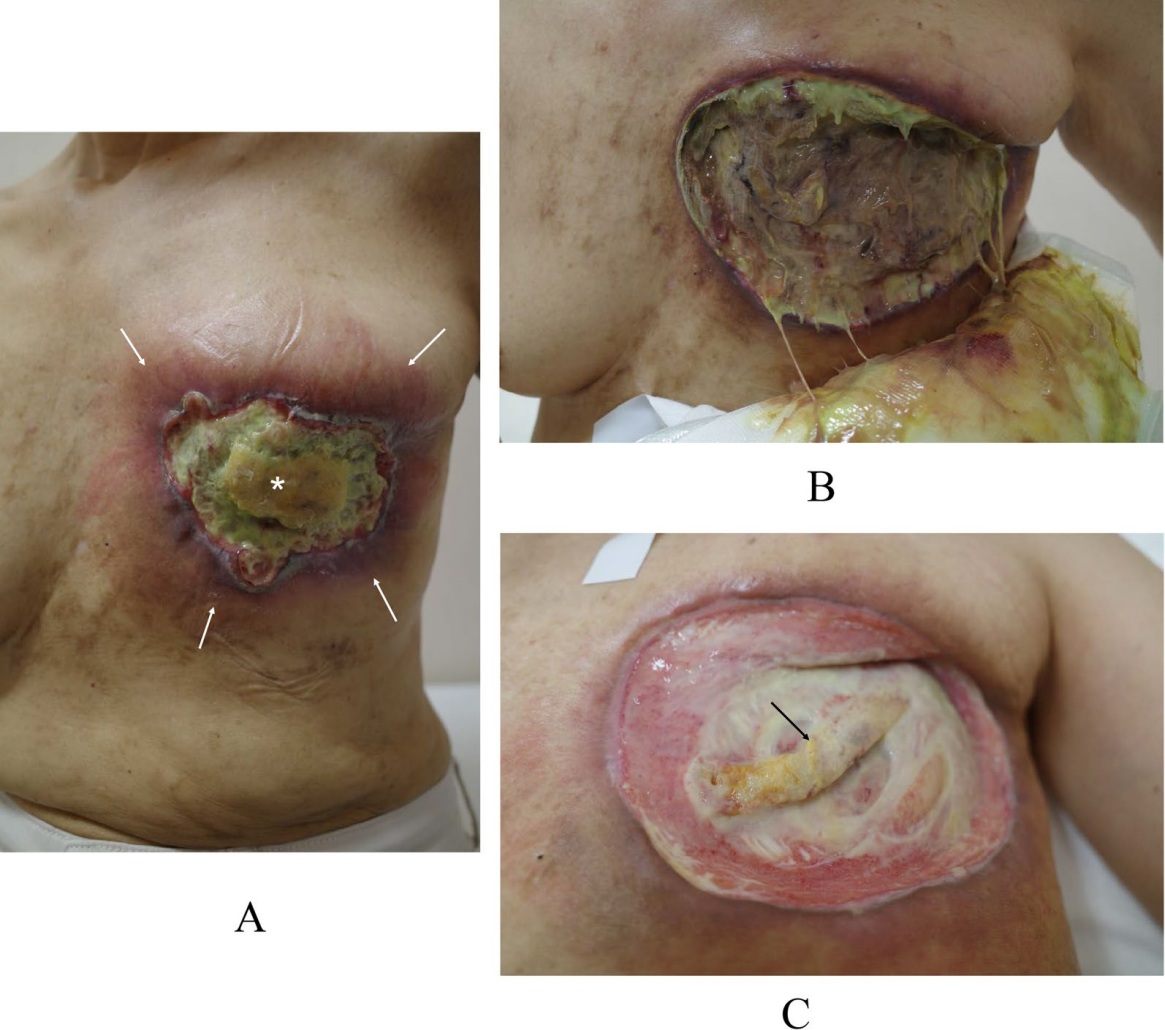
Local findings. **A** The left breast was completely necrotic with *Pseudomonas* *aeruginosa* infection (asterisk), and marked skin redness (arrows) was observed around the necrotic tissue. **B** Necrotomy caused total loss of the left breast and at least major part of the pectoral muscles. Necrotic tissue and massive purulent discharge were still observed just after the necrotomy. **C** Neither tumor nor mammary gland was observed on the left chest wall after the completion of bevacizumab and paclitaxel therapy for four months. Slightly elevated 4th rib (arrow) was seen in the center of the exposed chest wall

**Fig. 2:**
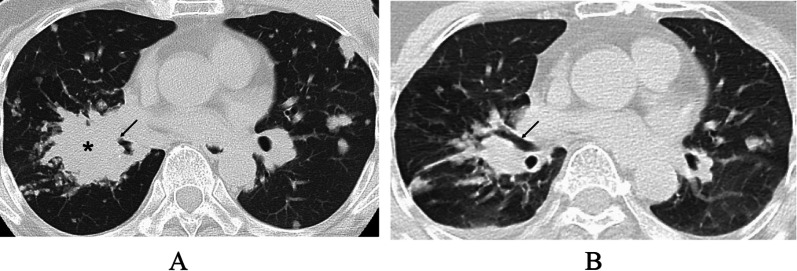
Computed tomography (CT) of the chest. **A** CT just before her first visit to our hospital showed multiple lung nodules. The largest lesion (asterisk) involved the right pulmonary hilum, causing stenosis of the bronchus intermedius (arrow). **B** Bevacizumab and paclitaxel therapy brought about marked regression of the lung metastatic foci and improvement of the bronchial stenosis (arrow)

**Fig. 3:**
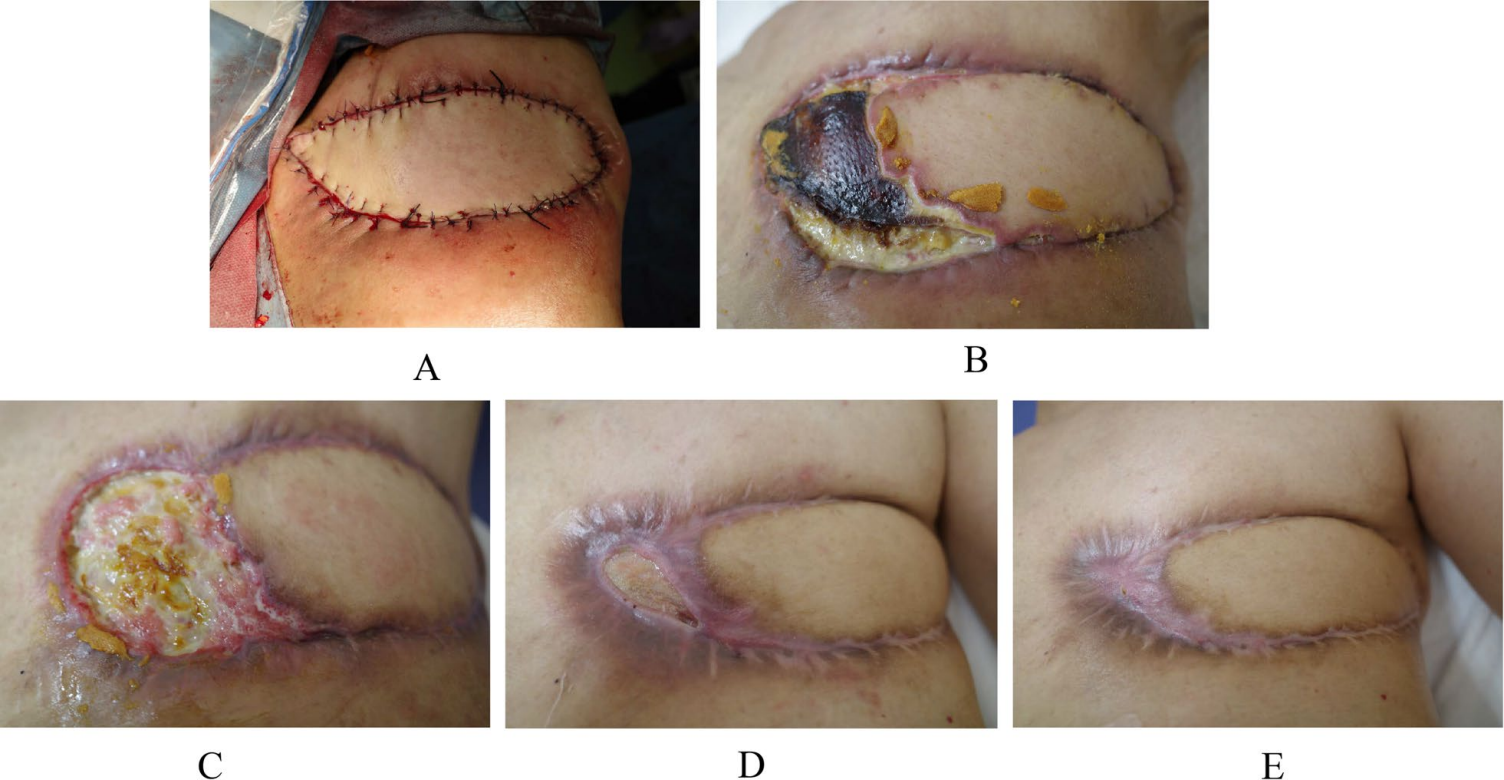
Post-operative local findings. **A** Skin defect was well covered with the latissimus dorsi musculocutaneous (LD-MC) flap. **B** Partial flap necrosis was observed in the distal part of the LD-MC flap. **C** Three months after operation, despite the large skin defect, no ribs and intercostal muscles were observed. **D** Eight months after operation, the skin defect became much smaller. **E** Eleven months after operation, complete skin healing was finally observed

A 66-year-old woman consulted a hospital due both to a large tumor in her left breast and presumed spinal metastasis-induced lower limb paresthesia. Core needle biopsy to the breast tumor showed atypical cells growing in a papillary fashion in the mucous lake, leading to the diagnosis of breast mucinous carcinoma. The tumor showed high estrogen receptor positivity, i.e., Allred score of 8, low growing potential, i.e., Ki-labeling index of 10%, and human epidermal growth factor receptor type 2 negativity. Onset of the paresthesia and the bleeding from the left breast cancer forced the attending physicians to treat the patient with tumorectomy of the intradural extramedullary tumor, 50 Gy radiotherapy to the left breast cancer, and systemic therapy using letrozole and palbociclib. Based on the request of the patient’s family, the patient was further referred to our hospital for the treatment of her metastatic breast cancer. At her first visit to our hospital, we found no paresthesia, no bleeding from the breast cancer, and total necrosis of the left breast with bacterial culture-proven *Pseudomonas* *aeruginosa* infection (Fig. 1a). Removal of the necrotic breast tissue resulted in direct exposure of the left ribs and intercostal muscles (Fig. 1b, c), markedly deteriorating quality of life of the patient due to the onset of severe chest pain. Computed tomography (CT) showed bilateral multiple lung metastases (Fig. 2a). The largest pulmonary nodule seemed to infiltrate the right pulmonary hilum. We, therefore, judged the lung metastases to be life-threatening and converted the systemic therapy from letrozole plus palbociclib to bevacizumab plus paclitaxel, fortunately leading to marked regression of the lung metastases (Fig. 2b). *Pseudomonas* *aeruginosa* infection continued even after the upfront therapies, i.e., removal of the necrotic left breast followed by bevacizumab-containing chemotherapy, in our hospital. After confirming marked regression of the multiple lung metastases, to improve quality of life of the patient, we decided to cover the exposed ribs and intercostal muscles with LD-MC flap for chest pain control and wound management. Although we did not perform a bacterial culture test just before surgery, we judged that *Pseudomonas* *aeruginosa* infection continued to some extent on the rib periosteum and intercostal muscle surface based on local macroscopic findings. Before operation we obtained full informed consent about possible total flap loss because no reports had been made at that time concerning the MC flap grafting to the overtly infected recipient site. To cover the skin defect, spindly shaped skin island, 22 × 6 cm in size, on the back was harvested with the LD muscle and was moved onto the skin defect area after resecting the exposed left 4th rib and removing the two swollen axillary lymph nodes (Fig. 3a). LD-MC flap grafting to the skin defect brought about immediate, i.e., when the patient became fully conscious after awaking from anesthesia, pain relief to the patient. The patient had required oral morphine and loxoprofen for pain control before surgery, but no longer needed pain relievers after surgery. Pathological evaluation of the sampled axillary lymph nodes showed no viable cancer cells in the mucous lakes widely prevailing in the nodes. Skin island showed no problems for 4 days just after the operation but thereafter gradually turned out to be edematous to ill-colored in the distal part, i.e., approximately 1/5 area, of the flap skin (Fig. 3b). The patient eventually developed partial skin necrosis of the LD-MC flap and required 11 months for complete wound healing under conservative wound management (Fig. 3c–e). The patient has been well on fulvestrant and palbociclib therapy for 14 months after palliative chest wall covering surgery with no progression of the lung metastases.

## Discussion

Main goal in treating metastatic breast cancer (MBC) is to alleviate or prevent uncomfortable symptoms induced by MBC [5]. Therefore, surgical intervention to the intradural extramedullary metastasis was done to the patient to improve the uncomfortable paresthesia, fortunately leading to successful recovery from the spinal cord injury. Another intervention was radiotherapy to the left breast to control the bleeding from cancer, leading to successful hemostasis on one hand. Radiotherapy, however, unfortunately caused total necrosis of the left breast on the other hand, causing unfavorable *Pseudomonas* *aeruginosa* infection to the necrotic breast tissue and severe chest pain due to the direct exposure of the chest wall after necrotomy. Details of 50 Gy radiotherapy to the left breast were unknown because the radiotherapy had been done in another hospital before the referral to our hospital. Radiation oncologists, however, should note this type of complication, i.e., total breast necrosis. In addition, breast oncologists should always take the Mohs’ chemosurgery [6], i.e., less invasive than radiotherapy, into consideration to control the bleeding from the breast.

The marked improvement of local infection might suggest that the *Pseudomonas* *aeruginosa* infection might have resolved before surgery. It, however, is extremely rare that the bacterial infection is cured while the skin defect remains. Both the periosteum and the muscle had a pale green color tone on the surface. It, therefore, seems reasonable to assume that *Pseudomonas* *aeruginosa* infection had persisted, although the bacterial load was likely to have decreased dramatically.

It is sometimes difficult for breast oncologists to judge whether the MBC is life-threatening or not. In this case, cancer spread to the major bronchus seemed to be life-threatening because pulmonary hilum involvement should shortly cause inevitable atelectasis, often leading to severe morbidity or even mortality. We, therefore, treated the patient with bevacizumab and paclitaxel for prompt tumor regression, successfully resulting in marked regression of the lung metastases with the bevacizumab-containing chemotherapy. It is often experienced that mucinous breast carcinoma leaves acellular mucin even after remarkably responding to some kind of anticancer therapy. This finding was actually observed in the pathological examination of the removed axillary lymph nodes in this case. In addition, judged by favorable clinical outcome after chemotherapy, at least major part of the regressed but still remaining lung foci may consist of acellular mucin.

To fulfill the large skin defect, we can generally use skin graft, skin substitutes [7], and MC flaps. Skin graft and skin substitutes are very useful to cover the skin defect but need careful recipient site preparation such as well vascularized wound bed, no necrotic or ischemic tissue, and no inflammation/infection. Neither skin graft nor skin substitutes, therefore, were suited for skin coverage due to the prolonged *Pseudomonas* *aeruginosa* infection on the irradiated and exposed chest wall. MC flap grafting, therefore, was the appropriate therapeutic option we could select in this case.

Concerning MC flap grafting in oncoplastic surgery, transverse rectus abdominis MC flap grafting has an advantage of large breast reconstruction and a disadvantage of blood flow instability. LD-MC flap has a disadvantage of limited volume transfer but has an advantage of stable blood flow. In fact, we found no ischemia or congestion of the transferred LD-MC flap at least for four days just after the operation. However, MC flap necrosis due to insufficient blood flow generally occurs at the distal part of the MC flap, which well corresponds to the flap necrosis site in this case. We, therefore, could not exclude the possibility of insufficient blood flow-induced necrosis of the LD-MC flap. To the best of our knowledge, no reports have been thus far made concerning the MC flap grafting to the infected recipient site. Chen et al. [8] reported surgical site Fusarium infection and partial necrosis, presumably due to the vessel occlusion with the formation of microemboli, of the MC flap after free anterolateral thigh MC flap grafting. Although we cannot precisely assess the etiology of flap necrosis in this case due to the lack of pathological evaluation of the necrotic tissue of the LD-MC flap, *Pseudomonas* *aeruginosa* infection may have had some adverse effect, e.g., microemboli, on MC flap blood flow. It, therefore, seems to be worth trying for breast surgeons to use anti-coagulant therapy such as heparin as soon as possible just after the MC flap grafting to the infected recipient site to prevent microemboli.

Breast cancer surgeries themselves consist of fundamentally aseptic procedures. Breast surgeons, therefore, rarely have the opportunity to perform surgery in the infected operative field and generally hesitate to graft some kind of MC flaps to the obviously infected surgical site due to the lack of surgical outcome information on such situation. Newly developed various anticancer agents, however, should further prolong the survival of MBC patients, highly suggesting the increased chances for breast surgeons to perform some kind of operations to the infected site for improving quality of life of the patient. Breast surgeons, therefore, should note that LD-MC flap can be a promising option to cover the skin defect even with obvious infection but should be managed with anti-coagulant therapy before overt clinical manifestation of flap change.

## Conclusions

This is the first case of LD-MC flap grafting to the infected recipient site. On applying LD-MC flap to the infected site, breast surgeons should consider the anti-coagulant therapy just after the operation to avoid the adverse effects of the infection.

## Data Availability

All data generated or analyzed during this study are included in this published article.

## Abbreviations

MC: Musculocutaneous
LD: Latissimus dorsi
CT: Computed tomography
MBC: Metastatic breast cancer
